# The Cost-Effectiveness of Monitoring Strategies for Antiretroviral Therapy of HIV Infected Patients in Resource-Limited Settings: Software Tool

**DOI:** 10.1371/journal.pone.0119299

**Published:** 2015-03-20

**Authors:** Janne Estill, Luisa Salazar-Vizcaya, Nello Blaser, Matthias Egger, Olivia Keiser

**Affiliations:** 1 Institute of Social and Preventive Medicine (ISPM), University of Bern, Bern, Switzerland; 2 Centre for Infectious Disease Epidemiology and Research, University of Cape Town, Cape Town, South Africa; University of Maryland School of Medicine, UNITED STATES

## Abstract

**Background:**

The cost-effectiveness of routine viral load (VL) monitoring of HIV-infected patients on antiretroviral therapy (ART) depends on various factors that differ between settings and across time. Low-cost point-of-care (POC) tests for VL are in development and may make routine VL monitoring affordable in resource-limited settings. We developed a software tool to study the cost-effectiveness of switching to second-line ART with different monitoring strategies, and focused on POC-VL monitoring.

**Methods:**

We used a mathematical model to simulate cohorts of patients from start of ART until death. We modeled 13 strategies (no 2^nd^-line, clinical, CD4 (with or without targeted VL), POC-VL, and laboratory-based VL monitoring, with different frequencies). We included a scenario with identical failure rates across strategies, and one in which routine VL monitoring reduces the risk of failure. We compared lifetime costs and averted disability-adjusted life-years (DALYs). We calculated incremental cost-effectiveness ratios (ICER). We developed an Excel tool to update the results of the model for varying unit costs and cohort characteristics, and conducted several sensitivity analyses varying the input costs.

**Results:**

Introducing 2^nd^-line ART had an ICER of US$1651-1766/DALY averted. Compared with clinical monitoring, the ICER of CD4 monitoring was US$1896-US$5488/DALY averted and VL monitoring US$951-US$5813/DALY averted. We found no difference between POC- and laboratory-based VL monitoring, except for the highest measurement frequency (every 6 months), where laboratory-based testing was more effective. Targeted VL monitoring was on the cost-effectiveness frontier only if the difference between 1^st^- and 2^nd^-line costs remained large, and if we assumed that routine VL monitoring does not prevent failure.

**Conclusion:**

Compared with the less expensive strategies, the cost-effectiveness of routine VL monitoring essentially depends on the cost of 2^nd^-line ART. Our Excel tool is useful for determining optimal monitoring strategies for specific settings, with specific sex-and age-distributions and unit costs.

## Introduction

The latest World Health Organization (WHO) guidelines recommend routine viral load monitoring for HIV-infected patients on antiretroviral therapy (ART) in resource-limited settings [1]. Routine viral load (VL) monitoring is the gold standard for detecting treatment failure and deciding when patients should switch to 2^nd^-line ART. VL monitoring may also support adherence and prevent HIV transmission, thus offering advantages beyond patient survival [2]. Most ART programs in resource-limited settings currently rely on CD4 or clinical monitoring [3], and the debate over the long-term benefit of routine VL monitoring still continues. It centers on the high cost of VL and on the logistical constraints that may make it infeasible to implement this recommendation [4–8].

We have shown that monitoring VL with a qualitative, moderately-sensitive POC VL test benefits the patient, and may be cost-effective compared with CD4 or clinical monitoring [5]. Rapid and affordable point-of-care (POC) tests for VL are already in development [9,10]. POC tests may improve on laboratory-based monitoring by simplifying the process. Patients could get same-day test results, adherence counseling, and/or make treatment decisions, all during the same visit. Though cheaper, POC tests may not be as accurate for diagnosis as sensitive and fully quantitative tests. Nevertheless, in settings where VL monitoring is still unavailable, POC tests may offer an affordable entry into VL monitoring.

The feasibility and cost-effectiveness of VL monitoring with a qualitative POC test depends on many factors, and these can vary substantially among settings. We built on our earlier mathematical simulation model of patients on ART, to create a flexible and user-friendly tool that allows users to evaluate the cost-effectiveness of a wide range of monitoring and switching strategies with varying assumptions.

## Methods

### Simulation model

**Fig 1:**
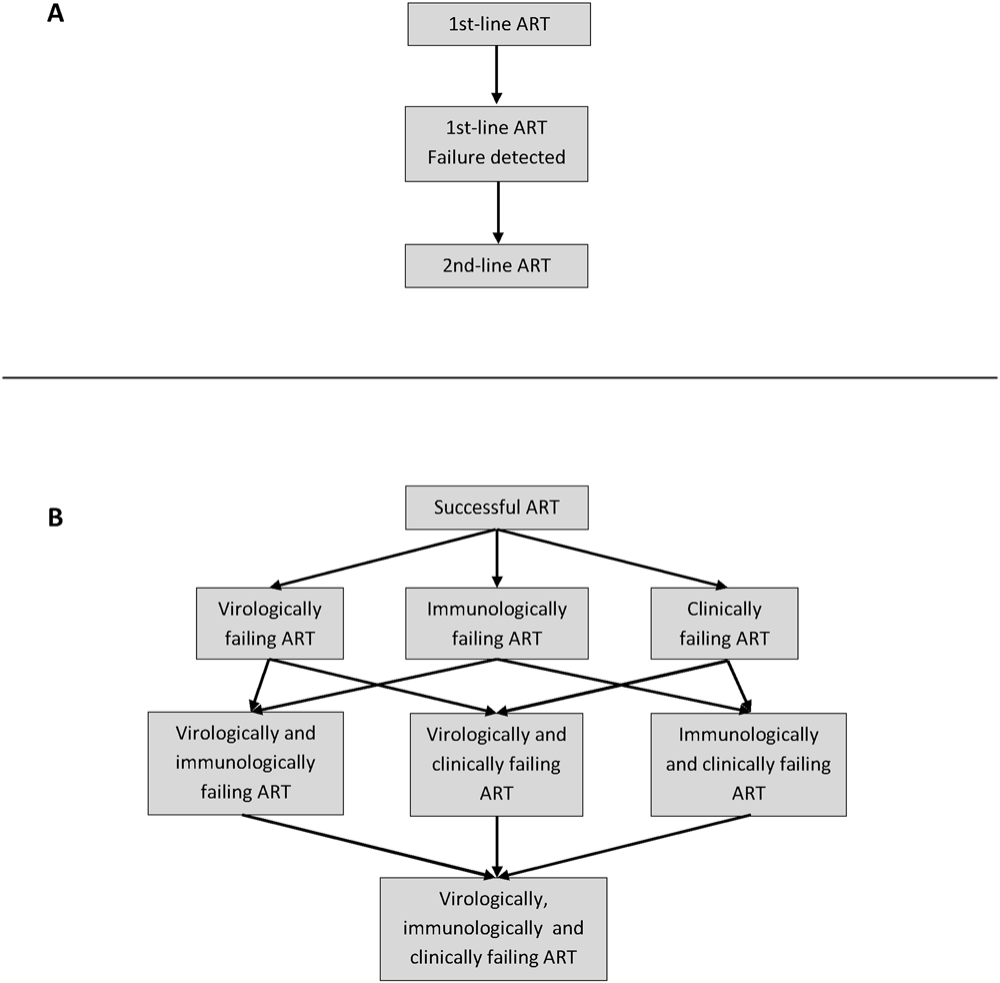
Progression of patients in the mathematical model. Panel A shows the progression of the patient’s treatment regimen and observed failure status. Within each compartment of panel A, the patient will proceed according to the underlying treatment progression shown in Panel B. The type of failure that can be detected depends on the monitoring strategy. After switching to second-line therapy, the patient will start either in the successful ART compartment (if he/she had no or concordant immunological/clinical failure) or in the clinical and/or immunological failure compartment (if he/she had a discordant failure of the corresponding type). See the main text for definitions of concordant and discordant failures.

We modeled cohorts of HIV-infected patients from ART initiation until death. We used three indicators to define the progression of HIV infection qualitatively: virological; immunological; and, clinical. All three indicators have two possible values: normal and failing. We assumed that all patients started in the normal virological, immunological and clinical stages. This represents the successful introduction of ART: VL decreases rapidly to undetectable values and remains suppressed; CD4 cell count increases; and, the patient will have no more clinical (WHO stage 3 or 4 defining) symptoms. The patient can proceed to virological failure at any time: this represents a rebound in VL to a detectable value of >1000 copies/ml (or, at the early stages of treatment, the failure to suppress). Progression to immunological failure represents a CD4 cell count decline to a level that meets the WHO immunological criteria of treatment failure [1]. Progression to clinical failure represents the occurrence of WHO stage 3 or 4 defining symptoms. Both immunological and clinical progression can occur either as a consequence of virological failure (“concordant”), or independent of the patient’s virological stage (“discordant”). Clinical progression also depends on the patient’s current immunological status. The parameterization of hazard functions related to disease progression was the same as in previous modeling studies [2,4,5], which were mostly based on two routine ART programs from the Cape Town area, Gugulethu and Khayelitsha. The characteristics of these cohorts were described in a previous publication [2]. The structure of the simulation model is presented in Fig. 1 and the key parameters are listed in Table 1.

**Table 1:** Key parameters related to disease progression.

Outcome	Source	Statistical model	Starting	Value (95% CI)	Dimension	Risk
**1) Time to virological failure**						
(a) First-line ART; second-line ART with immediate switch	Cohorts	Parametric Weibull	3 months from ART start/switch	0.57 (0.52–0.63)	Shape	3.4% fail by 1 year after ART start
				2.75 (2.29–3.31)	Scale (100 years)	
(b)Resistance penalty	[37]	*	n/a	0.05 (0.00–0.20)	Decrease in efficacy	n/a
**2) Time to immunological failure**						
(a) After virologic failure	Cohorts	Parametric exponential	Virological failure	0.08 (0.06–0.10)	Rate (years^-1^)	7.6% fail by 1 year after virologic failure
(b) Independently of virologic failure	Cohorts	Parametric Weibull	3 months from ART start	0.22 (0.20–0.25)	Shape	3.0% fail by 1 year after ART start
				5.46 (3.14–9.51)	Scale (10^6^ years)	
**3) Time to clinical failure**						
(a)Without virologic or immunologic failure	[6]	Parametric exponential	ART start	0.004	Rate (years^-§^)	0.4% fail by 1 year after ART start
(b) Extra hazard after immunologic failure	[6,38]	Cox regression	Immunologic failure	3.3	HR, constant over time	n/a
(c) Extra hazard after virologic failure	[6]	Cox regression	Virologic failure	2	HR, constant over time	n/a
**4) Time to death (HIV-related mortality)**						
(a) Observed mortality	Cohorts	No specific model (competing risk analysis)	ART start	**	n/a	6.5% die by 1 year after ART start
(b) Observed LTFU	Cohorts	No specific model (competing risk analysis)	ART start	**	n/a	4.0% lost by 1 year after ART start
(c) Mortality among LTFU	Analysis 4b, [39]	No specific model (theoretical calculation)	n/a	**	n/a	n/a
(d) HIV-related mortality	Analyses 4a-4c	Theoretical calculation, double Weibull***	ART start	0.88 (0.88–0.90)	Shape 1	8.8% die by 1 year after ART start
				0.35 (0.32–0.39)	Scale 1 (years)	
				1.00 (1.00–1.00)	Shape 2	
				64.60 (54.52–76.55)	Scale 2 (years)	
				0.08 (0.08–0.08)	Weight (1^st^ component)	
(a) Extra hazard after clinical failure	assumption	Cox regression	Clinical failure	2	HR, constant over time	n/a
(b) Extra hazard after immunologic failure	Cohorts	Cox regression	Immunologic failure	1.76 (1.16–2.68)	HR, constant over time	n/a
(c) Extra hazard after virologic failure	Cohorts	Cox regression	Virologic failure	1.26 (0.86–1.85)	HR, constant over time	n/a

The hazard of virological failure (1a) is applied for first-line ART as such, and for second-line ART together with a resistance penalty factor (1b) which depends on the time spent on failing first-line ART. Immunological failure can happen through two independent hazard functions: the other is applied only to patients on virologically failing first- or second-line ART (2a), the other for all patients irrespective of the virological status or ART regimen (2b). For clinical failure, the hazard function (3a) is used as such for patients without virological and immunological failures, and the hazard ratios (3b, 3c) are applied for patients with the corresponding failures. HIV-related mortality is calculated from a competing risk analysis of observed mortality (4a) and loss to follow-up (4b) as well as the expected mortality among lost patients (4c). The parametric hazard function for mortality (4d) is used as such for patients without virological, immunological or clinical treatment failure, and the hazard ratios (4e, 4f, 4g) are applied to patients with the corresponding failures.

CI, confidence interval; ART, antiretroviral therapy; HR, hazard ratio; LTFU, loss to follow-up; n/a, not applicable

* Relative decrease in second-line efficacy per year spent on failing first-line ART

** Observed mortality and LTFU rates on successful first-line ART were calculated from the data and used, together with background mortality and expected mortality among patients LTFU, to calculate the corrected HIV-related mortality for the cohort

*** Weighted sum of two Weibull distributions

The failing virological, immunological and clinical stages will persist, unless the patient switches to a 2^nd^-line ART regimen. When the patient switches, the failing virological as well as concordant failing immunological and clinical stages will return to normal. Failing discordant immunological and clinical stages will remain failing after switching. During 2^nd^-line therapy, the patient is again at risk of proceeding to the failing virological, immunological or clinical stage. The parameterization was the same as for 1^st^-line therapy, except that the risk of proceeding to virological failure was scaled up with a resistance penalty factor, which depended on the time the patient spent on virologically failing 1^st^-line therapy.

Mortality consists of two components: HIV-free background mortality and HIV-related mortality. We assumed the risk of mortality increased for patients in the failing virological, immunological or clinical stage. Although we assumed that all patients are retained in care from ART initiation until death, we accounted for the expected high mortality among patients lost to follow-up when we estimated the HIV-related mortality rates [4].

We considered a total of 13 monitoring and switching strategies (Table 2). With *clinical monitoring*, the treatment failure is observed at the next regular appointment after the patient proceeds to the advanced clinical stage, and the patient switches 3 months later. With *CD4 monitoring*, the failure is observed at the next appointment when the CD4 cell count is measured (which depends on the measurement frequency), and the patient switches 3 months later. Both clinical and CD4 monitoring are assumed to be fully specific and sensitive for detecting clinical or immunological failure. With *routine VL monitoring*, observing the failure depends on the definition of failure and the test itself. With a *laboratory VL test*, a failure is observed at the first monitoring appointment after the failing virological stage begins, and we assumed that CD4s are measured simultaneously. With a *qualitative POC VL test*, a failure may be observed at any visit, and the probability of detecting a failure depends on the detection limit of the test: we assumed it was 5000 copies/ml throughout the study. The test is repeated three months after observing the failure; if the failure is confirmed, the patient switches to 2^nd^-line ART. With *targeted VL monitoring*, only CD4 counts are measured routinely. A POC VL test is performed immediately if an immunological failure is detected. If this test is also positive, a second VL test is performed 3 months later.

**Table 2:** Monitoring strategies.

Strategy	Visits	CD4 tests	VL tests	Switching criteria
**1. No 2** ^**nd**^ **-line ART**				
1.1 No 2^nd^-line ART	every 3 months*	no	no	no
**2. Clinical monitoring**				
2.1 Clinical monitoring	every 3 months	no	no	WHO clinical criteria
**3. CD4-based monitoring**				
3.1 Irregular CD4 monitoring 6m	every 3 months*	every 6 months**	no	2x WHO immunological criteria
3.2 CD4 monitoring 24m	every 3 months*	every 24 months	no	2x WHO immunological criteria
3.3 CD4 monitoring 12m	every 3 months*	every 12 months	no	2x WHO immunological criteria
3.4 CD4 monitoring 6m	every 3 months*	every 6 months	no	2x WHO immunological criteria
3.5 CD4 6m + tVL monitoring	every 3 months*	every 6 months	POC after CD4 failure	WHO immunological criteria + 2x VL≥5000 copies/ml
**4. Point-of-care viral load monitoring**				
4.1 POC-VL monitoring 24m	every 3 months*	no	POC every 24 months	2x VL≥5000 copies/ml
4.2 POC-VL monitoring 12m	every 3 months*	no	POC every 12 months	2x VL≥5000 copies/ml
4.3 POC-VL monitoring 6m	every 3 months*	no	POC every 6 months	2x VL≥5000 copies/ml
**5. Laboratory-based viral load monitoring**				
5.1 Lab VL monitoring 24m	every 3 months*	every 24 months*	Lab every 24 months	2x VL≥1000 copies/ml
5.2 Lab VL monitoring 12m	every 3 months*	every 12 months*	Lab every 12 months	2x VL≥1000 copies/ml
5.3 Lab VL monitoring 6m	every 3 months*	every 6 months*	Lab every 6 months	2x VL≥1000 copies/ml

ART, antiretroviral therapy; VL, viral load; tVL, targeted viral load; m, monthly; POC, point-of-care; Lab, laboratory-based

POC VL tests are assumed to be qualitative with a detection limit of 5000 copies/ml; lab-VL tests are assumed to be fully quantitative

2x = second confirmatory measurement 3 months after first observation needed

* The information from these visits/tests is not used to decide about switching to second-line

** The probability of having a test is 50%

We modeled two scenarios. In *Scenario A*, we assumed that the risk of treatment failure did not depend on the monitoring and switching strategy, and thus, for all strategies, we used the rates estimated from the South African data (where VL is monitored regularly). In *Scenario B*, we assumed that VL monitoring can prevent treatment failure, and that the South African rates underestimate the risk in strategies where VL monitoring is unavailable. We assumed this because routine viral load monitoring can detect poor adherence. Many patients with a detectable viral load can re-suppress viral load on first-line ART, after an adherence intervention [11–14]. Since poor adherence is a major predictor of treatment failure [15,16], routine viral load monitoring may prevent treatment failures caused by poor adherence. For these strategies, we assumed that the hazard would be twice as high across the entire follow-up period.

The model was constructed in three steps. In the first step, we simulated cohorts of 100,000 patients for all 13 monitoring and switching strategies, without baseline characteristics or background mortality. We implemented the model using ‘gems’, an R package for generalized multistate simulation models [17,18]. ‘Gems’ models disease progression as a series of events (e.g., diagnosis, treatment and death) that can be displayed in a directed acyclic graph (DAG). The vertices correspond to disease states and the directed edges represent events. Similar models that use the package ‘gems’ or a similar algorithm have been published elsewhere [2,4,5,19]. For strategies without routine VL monitoring we modeled two cohorts with different failure rates (scenarios A and B). This resulted in a total of 20 cohorts.

In the second step, these cohorts were updated to account for differences in background mortality. Thirty-two copies of each of the 20 cohorts were created, for the different sexes (male and female), baseline age groups (15–24, 25–34, 35–44 and 45–54 years), and four different scenarios of background mortality. In the first three scenarios, the background mortality rates represent the overall HIV-free mortality in the general populations of Malawi and Zimbabwe [20], and in Africans in the Western Cape [21]. In the fourth scenario, we assumed that the HIV-free life expectancy from birth would be 75 years for all patients. Each patient was assigned an age, sampled from a uniform distribution within the given range. A time of HIV-unrelated death was sampled for each patient, based on the gender, baseline age and the background mortality rate.

In the third step, we analyzed the outcomes of interest. The main outcomes were disability-adjusted life-years (DALY) lost to HIV, total cost, and cost-effectiveness ratios of the intervention compared to current practice as well as to the next less expensive strategy (incremental cost-effectiveness ratio, ICER). Definitions are given in S1 Text.

### Excel tool

We developed an Excel spreadsheet tool to adapt the model outputs to specific scenarios. The Excel table contains all outputs of the simulation and presents the results according to the scenario defined by the user. The user can vary the following input variables continuously (Table 3): size of cohort; unit costs for clinic visit, VL and/or CD4 test, one year of 1^st^- and 2^nd^-line ART; and, disability weight of symptomatic and asymptomatic HIV. The user can specify the age and gender distribution of the cohort by giving proportions for each of the eight age and gender groups. Finally, the user can specify the failure rate scenario (A or B), background mortality (Malawi, Zimbabwe, Western Cape, or constant life expectancy 75 years) and discounting (0%, 1%, 2%, 3%, 4%, or 5%). The main results are then updated based on the assumptions. The Excel table also shows the ICERs, and graphically presents the costs and averted DALYs of each scenario.

**Table 3:** Input parameters that can be varied using the Excel tool.

Input	Value for main analysis	Values for sensitivity analyses	Values included in the Excel tool
**Costs**			
Visit	Not included	Not included	0 to infinity
CD4 test	US$ 5	US$ 2	0 to infinity
POC viral load test	US$ 10	US$ 5, US$ 7, US$ 15	0 to infinity
Laboratory viral load test	US$ 10	US$ 5, US$ 7, US$ 15	0 to infinity
1^st^-line ART per year	US$ 99	US$ 55, US$ 128	0 to infinity
2^nd^-line ART per year	US$ 280	US$ 140, US$ 210, US$ 350	0 to infinity
**Disability coefficients**			
Asymptomatic HIV	0.135	-	0 to 1
Symptomatic HIV	0.369	-	0 to 1
**Size of cohort**			
Size of cohort	1	-	1 to infinity
**Discounting**			
Annual discounting rate	3%	0%	0%, 1%, 2%, 3%, 4%, 5%
**Age and gender distribution**			
Proportion of each age-gender group	1% M15–24; 8% F15–24; 12% M25–34; 35% F25–34; 14% M35–45; 18% F35–44; 6% M45–54; 6% F45–54	-	0 to 100% in any group, summing to 100%
**Background mortality**			
HIV-unrelated mortality	ASSA2008 (Africans in Western Cape)	-	GBD Malawi; GBD Zimbabwe; ASSA2008 Africans in Western Cape; 75 years*
**Virological failure rate**			
Virological failure rate in strategies without viral load monitoring (1.1–3.5)	Identical to strategies 4.1–5.3 (see Table 2 for the parameters)	Twice as high compared to strategies 4.1–5.3**	Identical or twice as high compared to strategies 4.1–5.3

ART, antiretroviral therapy; US$, US dollar; M, male; F, female; GBD, Global Burden of Disease study; ASSA2008, ASSA2008 model

* Every simulated patient dies at the age of 75 if the effect of HIV is not accounted for

** This analysis is presented as the second main analysis, not a sensitivity analysis

We present the results in this manuscript for a set of input parameters (Table 3). We assumed the costs of 1^st^-line ART were US$99/year and 2^nd^-line ART were US$280/year. VL tests (both POC and laboratory-based) were assumed to cost US$10, and CD4 tests US$5. We did not consider the cost of clinic appointments. Disability coefficients were 0.135 for asymptomatic HIV and 0.369 for symptomatic HIV. The disability coefficient for symptomatic HIV was the product of the coefficients of asymptomatic HIV and tuberculosis, the most common opportunistic infection [22]. All results are presented per one patient. Costs and DALYs are discounted annually by 3%. Two separate analyses were conducted: one in which virological failure rates were identical (Scenario A); and one in which it was twice as high in strategies without routine VL monitoring than in strategies with routine VL monitoring (Scenario B). We also present 10 sensitivity analyses in the S2 Text and S1 Table, in which the unit costs of tests and ART and the discounting rate were varied.

## Results

### Excel tool

The Excel tool is presented in S1 Excel File.

### Scenario A: Failure rate is identical in all monitoring and switching strategies

In the absence of 2^nd^-line and monitoring, the average lifetime cost of ART was US$1419 per patient (Table 4). On average, each patient lost 7.3 DALYs to HIV. Adding 2^nd^-line ART with clinical monitoring (2.1) increased the costs to US$1563 per patient and averted 0.09 DALYs. The costs of strategies with CD4 monitoring ranged from US$1690 (3.2) to US$1812 (3.5). CD4 monitoring averted 0.05 to 0.09 DALYs more than clinical monitoring: the benefit was higher for Strategies 3.1 and 3.5 than for Strategies 3.2, 3.3 and 3.4. Targeted VL monitoring (3.5) cost, on average, an additional US$12 per patient, but saved about US$61 per patient for ART over the same strategy without VL tests (3.4) by not switching of patients with suppressed VLs. The total costs for strategies with VL monitoring ranged from US$1840 (4.1) to US$2216 (5.3). VL monitoring averted 0 to 0.05 DALYs more than the most effective CD4 monitoring strategy (3.5), and 0.09 to 0.14 more than clinical monitoring (2.1). There were no clear differences between POC and laboratory-based VL monitoring in the number of averted DALYs. The POC VL strategy with most frequent (every 6 months) monitoring (4.2) averted slightly fewer DALYs than POC VL monitoring every 12 months (4.3).

**Table 4:** Model outcomes: main analysis assuming the treatment failure rate to be identical in all strategies.

Strategy	No 2^nd^-l.	Clinical	CD4 monitoring					POC-VL monitoring			Lab-VL monitoring		
	**1.1**	**2.1**	**3.1**	**3.2**	**3.3**	**3.4**	**3.5**	**4.1**	**4.2**	**4.3**	**5.1**	**5.2**	**5.3**
Life-years													
Healthy life-years left	19.5	19.5	19.5	19.5	19.5	19.5	19.5	19.5	19.5	19.5	19.5	19.5	19.5
Life-years on 1st-line ART	14.3	13.7	13.3	13.3	13.2	13.2	13.6	12.7	12.7	12.5	12.8	12.7	12.7
Life-years on 2nd-line ART	0.0	0.8	1.3	1.2	1.3	1.3	0.9	1.8	1.9	2.0	1.8	1.9	1.9
Life-years without symptoms	13.6	13.8	13.8	13.8	13.8	13.8	13.8	13.9	13.9	13.9	13.9	13.9	13.9
Life-years with symptoms	0.7	0.7	0.7	0.7	0.7	0.7	0.7	0.7	0.7	0.7	0.7	0.7	0.7
Life-years lost to HIV	5.2	5.1	5.0	5.0	5.0	5.0	5.0	5.0	4.9	5.0	5.0	5.0	4.9
Disability-weighted life-years	2.1	2.1	2.1	2.1	2.1	2.1	2.1	2.1	2.1	2.1	2.1	2.1	2.1
***DALYs lost to HIV***	***7*.*3***	***7*.*2***	***7*.*1***	***7*.*1***	***7*.*1***	***7*.*1***	***7*.*1***	***7*.*1***	***7*.*1***	***7*.*1***	***7*.*1***	***7*.*1***	***7*.*1***
**Costs**													
Cost of 1^st^-line ART	1419	1353	1313	1313	1306	1304	1347	1261	1254	1238	1263	1256	1255
Cost of 2^nd^-line ART	0	211	351	341	363	365	259	507	534	563	499	521	530
Cost of diagnostic tests	0	0	71	36	72	143	156	73	147	292	107	216	431
***Total costs***	***1419***	***1564***	***1735***	***1690***	***1741***	***1812***	***1761***	***1840***	***1935***	***2094***	***1869***	***1993***	***2216***
**Cost-effectiveness**													
***CER compared to 1*.*1***	***l/e***	***1651***	***1877***	***1967***	***2243***	***2959***	***1910***	***2122***	***2304***	***3775***	***2359***	***2844***	***3585***
***ICER***	***l/e***	***1651***	***2123***	***w/d***	***s/d***	***s/d***	***2418***	***w/d***	***3872***	***s/d***	***s/d***	***s/d***	***s/d***

Please see Table 2 for a detailed description of all monitoring strategies. All costs are given in US$ and cost-effectiveness ratios in US$ per DALY averted.

POC-VL, point-of-care viral load; lab-VL, laboratory-based viral load; ART, antiretroviral therapy; DALY, disability-adjusted life-year; CER, cost-effectiveness ratio; ICER, incremental cost-effectiveness ratio; l/e, least expensive and least effective strategy; w/d, weakly dominated; s/d, strongly dominated.

**Fig 2:**
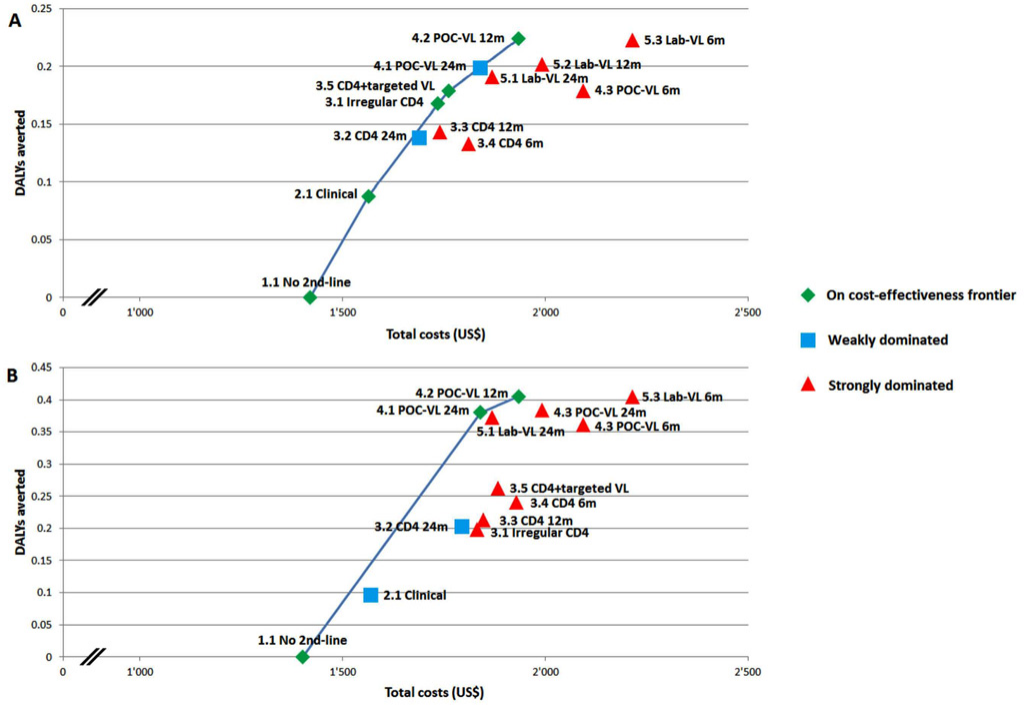
Cost-effectiveness of different monitoring strategies for antiretroviral therapy. Panel A presents Scenario A (failure rate identical in all monitoring strategies). Panel B presents Scenario B (failure rate twice as high in strategies without compared to strategies with routine viral load monitoring). Cost and DALYs averted are presented per one patient for the duration of ART. Please see Table 2 for a detailed description of the monitoring strategies.

No 2^nd^-line (1.1), clinical monitoring (2.1), irregular CD4 monitoring every 6 months (3.1), CD4 monitoring every 6 months with targeted VL monitoring (3.5), and POC VL monitoring every 12 months (4.2) were on the cost-effectiveness frontier (Fig. 2A). The cost-effectiveness ratio of clinical monitoring alone (2.1) compared with no 2^nd^-line (1.1) was US$1651/DALY averted. Compared with clinical monitoring alone (2.1), CD4 monitoring averted a DALY at costs betweenUS$2123 (3.1) and US$5488 (3.5). VL monitoring strategies prevented a DALY at costs between US$3478 (4.1) and US$33515 (4.3) as compared with Strategy 3.1 or US$2494 (4.1) to US$813 (4.3) as compared with Strategy 2.1.

### Scenario B: Failure rate is twice as high without VL monitoring as with VL monitoring

When only 1^st^-line ART was available (1.1), the average lifetime cost of ART was US$1401 per patient (Table 5). On average, each patient lost 7.5 DALYs to HIV. When 2^nd^-line ART with clinical monitoring was added (2.1), this increased the costs to US$1569 per patient and averted 0.10 DALYs. CD4 monitoring strategies cost between US$1794 (3.2) and US$1929 (3.4). CD4 monitoring averted 0.10 to 0.12 DALYs more than clinical monitoring: the benefit increased with monitoring frequency and was highest for targeted VL monitoring. Targeted VL monitoring (3.5) cost, on average, an additional US$13 per patient, but ART cost about US$58 less per patient than in Strategy 3.4, because patients with suppressed VLs did not need to be switched. The results of strategies that used VL monitoring (4.1 to 5.3) are the same as those in Scenario A.

**Table 5:** Model outcomes: main analysis assuming that treatment failure rate is twice as high in strategies without routine viral load monitoring (1.1 to 3.5) as in strategies with routine viral load monitoring due to improved adherence.

Strategy	No 2^nd^-l.	Clinical	CD4 monitoring					POC-VL monitoring			Lab-VL monitoring		
	**1.1**	**2.1**	**3.1**	**3.2**	**3.3**	**3.4**	**3.5**	**4.1**	**4.2**	**4.3**	**5.1**	**5.2**	**5.3**
**Life-years**													
Healthy life-years left	19.5	19.5	19.5	19.5	19.5	19.5	19.5	19.5	19.5	19.5	19.5	19.5	19.5
Life-years on 1^st^-line ART	14.2	13.3	12.5	12.5	12.4	12.4	12.8	12.7	12.7	12.5	12.8	12.7	12.7
Life-years on 2^nd^-line ART	0.0	0.9	1.9	1.9	2.0	2.0	1.7	1.8	1.9	2.0	1.8	1.9	1.9
Life-years without symptoms	13.3	13.5	13.6	13.6	13.7	13.7	13.7	13.9	13.9	13.9	13.9	13.9	13.9
Life-years with symptoms	0.8	0.7	0.7	0.7	0.7	0.7	0.7	0.7	0.7	0.7	0.7	0.7	0.7
Life-years lost to HIV	5.4	5.3	5.2	5.1	5.1	5.1	5.1	5.0	4.9	5.0	5.0	5.0	4.9
Disability-weighted life-years	2.1	2.1	2.1	2.1	2.1	2.1	2.1	2.1	2.1	2.1	2.1	2.1	2.1
***DALYs lost to HIV***	***7*.*5***	***7*.*4***	***7*.*3***	***7*.*3***	***7*.*2***	***7*.*2***	***7*.*2***	***7*.*1***	***7*.*1***	***7*.*1***	***7*.*1***	***7*.*1***	***7*.*1***
**Costs**													
Cost of 1^st^-line ART	1401	1320	1234	1237	1228	1228	1263	1261	1254	1238	1263	1256	1255
Cost of 2^nd^-line ART	0	249	527	521	547	558	466	507	534	563	499	521	530
Cost of diagnostic tests	0	0	70	36	72	143	156	73	147	292	107	216	431
***Total costs***	***1401***	***1569***	***1831***	***1794***	***1848***	***1929***	***1884***	***1840***	***1935***	***2094***	***1869***	***1993***	***2216***
**Cost-effectiveness**													
***CER compared to 1*.*1***	***l/e***	***1766***	***2168***	***1947***	***2102***	***2199***	***1849***	***1156***	***1317***	***1924***	***1258***	***1545***	***2019***
***ICER***	***l/e***	***w/d***	***s/d***	***w/d***	***s/d***	***s/d***	***s/d***	***1156***	***3715***	***s/d***	***s/d***	***s/d***	***s/d***

Please see Table 2 for a detailed description of all monitoring strategies. All costs are given in US$ and cost-effectiveness ratios in US$ per DALY averted.

POC-VL, point-of-care viral load; lab-VL, laboratory-based viral load; ART, antiretroviral therapy; DALY, disability-adjusted life-year; CER, cost-effectiveness ratio; ICER, incremental cost-effectiveness ratio; l/e, least expensive and least effective strategy; w/d, weakly dominated; s/d, strongly dominated.

Except for the no 2^nd^-line strategy (1.1), only POC VL monitoring every 24 (4.1) and 12 months (4.2) were on the cost-effectiveness frontier (Fig. 2B). Clinical monitoring was weakly dominated by POC VL monitoring every 24 months. Strategies based on CD4 monitoring cost about as much as those that used VL monitoring, but averted fewer DALYs. The cost-effectiveness ratio of clinical monitoring alone (2.1) compared with no 2^nd^-line (1.1) was US$1766/DALY averted. Compared with clinical monitoring alone (2.1), CD4 monitoring averted a DALY at costs between US$1896 (3.5) and US$2540 (3.1). VL monitoring strategies prevented a DALY at costs between US$48 (4.1) and US$1875 (5.3) as compared with Strategy 3.1 or US$951 (4.1) and US$2097 (5.3) as compared with Strategy 2.1.

### Sensitivity analyses

The results of the sensitivity analyses are presented in detail in Table 6 and S2 Text and S2 to S11 Tables. Of note is the dependence of the benefit of targeted routine VL monitoring on the ratio of the costs between 1^st^- and 2^nd^-line regimens. If the prices of 1^st^- and 2^nd^-line regimens were similar, targeted VL monitoring was dominated; if 2^nd^-line ART was assumed to be substantially more expensive, targeted VL monitoring was on the cost-effectiveness frontier.

**Table 6:** Cost-effectiveness of 12-monthly point-of-care viral load monitoring (strategy 4.2) compared with clinical monitoring (strategy 2.1), assuming that the risk of virological failure is either identical in both strategies (Scenario A) or twice as high with viral load monitoring than with clinical monitoring (Scenario B).

Analysis	Varied input value	Cost-effectiveness ratio (US$/DALY averted)	
		Scenario A	Scenario B
Main		2723	1180
VL1	Cost of VL test: US$7	2400	1037
VL2	Cost of VL test: US$5	2184	942
VL3	Cost of VL test: US$15	3262	1417
FL1	Cost of 1^st^-line ART: US$55/year	3045	1275
FL2	Cost of 1^st^-line ART: US$128/year	2511	1117
SL1	Cost of 2^nd^-line ART: US$210/year	2131	950
SL2	Cost of 2^nd^-line ART: US$140/year	1539	720
SL3	Cost of 2^nd^-line ART: US$350/year	3315	1409
DI1	No discounting	2015	892

All performed sensitivity analyses are listed in S1 Table and their full results in S2 to S11 Tables of the Supplementary Material. The results of Analysis CD1 are not shown since the cost of CD4 test does not influence either strategy. The input values used in the main analysis are shown in Table 3.

## Discussion

We simulated cohorts of patients who initiated ART under 13 different ART monitoring and switching strategies. VL monitoring was slightly more effective than CD4 monitoring (in particular if we assumed that VL monitoring also reduces the risk of treatment failure). CD4 monitoring was more effective than clinical monitoring, and clinical monitoring was more effective than 1^st^-line ART only. However, differences in the effectiveness of any two strategies were all below 1 DALY per patient. We observed no clear difference between monitoring strategies that measured at different intervals, or between VL monitoring that used fully quantitative, highly specific and sensitive laboratory tests or those that used a qualitative POC test. The cost-effectiveness of POC VL monitoring clearly improved if we reduced the gap between prices of 1^st^- and 2^nd^-line ART (Table 6).

Across our analyses, 12-month routine POC VL monitoring was on the cost-effectiveness frontier. The cost-effectiveness ratio of this strategy compared to clinical monitoring varied between US$700 and US$3300 per DALY averted. Cost-effectiveness improved if we assumed that VL monitoring reduces the risk of failure, and when the price of 2^nd^-line ART and 1^st^-line ART were close. The cost-effectiveness ratio of US$700 per DALY averted was reached if 2^nd^-line costs were reduced to minimum and we assumed that routine VL monitoring prevents failure. If we define a cost-effective intervention as having a cost-effectiveness ratio of less than 3 times the local per-capita gross domestic product, a cost-effectiveness ratio of US$700 per DALY averted can be considered cost-effective in any country [23,24]. We found no major differences between fully quantitative laboratory-based VL monitoring with CD4 tests, and qualitative POC VL monitoring. The potential disadvantage of a POC test that we assumed was the possibility of “false positive” switches to 2^nd^-line ART, i.e. switching patients who do not have a persistent detectable viral load. For example, if a patient has two successive detectable viral load values, and if the exact values of the VLs and the CD4 cell count are known, the clinician may be able to distinguish a patient failing therapy and a patient with blips, poor adherence, measurement errors, etc. The qualitative test only gives a positive or negative result, upon which a decision must be based. Although we did not find differences between laboratory-based and POC VL monitoring overall, increasing the frequency of POC VL monitoring from 12 to 6 months slightly decreased, rather than increased life-years. This was not the case for laboratory-based VL monitoring. We think this is caused by false positive failures with POC tests: increasing the measurement frequency increases the number of patients who switch unnecessarily, and this may affect their future treatment options.

The cost-effectiveness of targeted VL monitoring varied changed with the input parameters. We found that if the price of 2^nd^-line ART is considerably higher than for 1^st^-line ART, and if we assume no additional benefits for routine VL monitoring, it may be cost-effective to conduct routine CD4 monitoring with targeted VL testing. This strategy uses routine CD4 tests to detect patients who may be on a failing 1^st^-line regimen. Patients are then given VL tests to confirm their status. This strategy reduces the total costs by restricting use of 2^nd^-line ART to patients who need it most (those with low CD4 cell counts and a high risk of mortality), and by not switching patients with suppressed VLs. However, if we assume that routine VL monitoring can also reduce the risk of failure, for example, or if the cost difference between the regimens is small, routine VL monitoring is preferable.

Optimal monitoring strategy has been investigated in a number of mathematical modeling studies. Walensky *et al* published a review of five modeling studies that assessed the cost-effectiveness of different strategies for monitoring HIV-infected patients on ART [25]. Four of the models investigated VL monitoring [6,26–28]. After the review was published, similar modeling studies have appeared [29,30]. A recent article [31] systematically compared three models. All these studies suggest that VL monitoring may moderately improve the outcomes of ART programs, but cost-effectiveness estimates vary substantially between them. The variance may be caused by the different assumptions in the input values. We believe our study is the first to include a tool that allows users to easily vary input values.

### Limitations

Our analysis has several limitations. First, we only included time on ART. Diagnostic tests, and, in particular, CD4 cell counts, are usually recommended for monitoring patients before ART is initiated. If assessment of ART eligibility continues to depend on CD4 cell counts, CD4 monitoring will continue to be important. However, there is a growing tendency towards simpler rules for ART initiation, such as “Option B+”, in which all pregnant and breastfeeding women start lifelong ART [32], or universal “test and treat” [33]. We anticipate that, in many settings, there will be a decreased need to measure CD4 cell count to assess eligibility for ART. However, to estimate the true costs of CD4 testing, the use of CD4 cell measurements for purposes other than on-treatment monitoring must be taken into consideration. The usefulness of monitoring immune response by CD4 cell measurement during the first year of ART should also be evaluated.

We did not include loss to follow-up (LTFU). High rates of LTFU are a serious problem in most ART programs in resource-limited settings [34]. LTFU is a combination of unregistered deaths, unregistered transfers, and cases in which a patient has stopped ART [35]. We took the effect of LTFU on mortality into account in the parameterization of mortality. Patients who transferred to another ART clinic can be expected to take ART as recommended. We did not take include the effect of patients who stopped ART. POC monitoring may offer advantages over laboratory-based monitoring by making it easier to manage patients and by shortening wait times. This may, in turn, improve retention [36], and is another argument for POC monitoring, but it is not studied in the current analysis.

We did not model onward transmission. VL monitoring reduces the time the patient spends on a failing regimen. VL monitoring can prevent about 30% of transmissions from patients on treatment [2,5], and this may substantially improve the cost-effectiveness of VL monitoring. Therefore, the long-term population-level benefits of VL monitoring may be larger than we estimated.

We assumed that laboratory-based VL monitoring, together with CD4 tests, is 100% sensitive and specific to detect true treatment failure. Although this is a simplifying assumption, we wanted to include two distinct types of viral load test: one of these is as accurate as possible, and one has a lower sensitivity and specificity. The POC tests were assumed to be neither fully sensitive nor specific to detect true treatment failure. The users of the Excel tool can therefore choose either laboratory-based or POC viral load monitoring, depending on the diagnostic capacities of the viral load test they want to investigate. We also assumed CD4 tests are fully sensitive and specific. But since CD4 cell count poorly predicts true treatment failure, this assumption should not have much effect on the results.

The Excel tool allows users to vary input costs and several other input parameters. However, parameters related to disease progression cannot be changed in the Excel tool. Most key parameters for HIV progression are based on two large ART cohorts from the Cape Town area: Gugulethu and Khayelitsha. The results of the model reflect the characteristics of these routine ART programs, typical of southern Africa: the majority of patients are women, and most patients start ART with low CD4 cell counts and advanced clinical symptoms. We believe the results of our model can be generalized widely for ART programs in sub-Saharan Africa. However, there are also important differences between the cohorts we drew on, and others: the Cape Town cohorts had access to frequent laboratory measurements, and there is a continuous tendency to start ART earlier. Our model may not be able to catch all site-level characteristics of the different settings.

## Conclusion

POC VL testing appears to be a promising alternative for routinely monitoring ART in resource-limited settings, especially if we assume that viral load monitoring can prevent treatment failure by improving adherence, and that the gap between prices of 1^st^- and 2^nd^-line ART can be decreased. Under these conditions, VL monitoring every 12 or 24 months, with an affordable qualitative test, does not considerably increase the costs above those of CD4 monitoring. POC VL testing may offer the same benefit as frequently monitoring VL with a quantitative test. Routine VL monitoring may also have benefits beyond more accurate detection of treatment failure; it may for example be able to prevent treatment failure by improved adherence. Targeted VL monitoring, based on routine CD4 monitoring, may be an option if these potential benefits are small, if 2^nd^-line ART remains substantially more expensive than 1^st^-line ART, and if it is expected that 2^nd^-line ART cannot be provided for everyone failing virologically. Special attention should be paid to the price of 2^nd^-line ART, which we expect will play a more substantial role than the price of the diagnostic tests. To make routine viral load monitoring cost-effective, efforts must be made to drop the costs of 2^nd^-line antiretroviral drugs. The optimal monitoring and switching strategy for each setting depends substantially on factors such as the unit costs. Our Excel tool allows researchers and policy-makers to vary important cost and population structure parameters and may be a valuable tool for developing local monitoring guidelines.

## Supporting Information

S1 Excel File Excel tool for adapting the model outputs to specific scenarios. (XLSX)

S1 Table List of sensitivity analyses. (DOCX)

S2 Table Model outcomes: sensitivity analysis VL1 assuming that the cost of a viral load test is US$7. (DOCX)

S3 Table Model outcomes: sensitivity analysis VL2 assuming that the cost of a viral load test is US$5. (DOCX)

S4 Table Model outcomes: sensitivity analysis VL3 assuming that the cost of a viral load test is US$15. (DOCX)

S5 Table Model outcomes: sensitivity analysis CD1 assuming that the cost of a CD4 test is US$2. (DOCX)

S6 Table Model outcomes: sensitivity analysis FL1 assuming that the annual cost of 1^st^-line therapy is US$55.< /SI_Caption>
(DOCX)

S7 Table Model outcomes: sensitivity analysis FL2 assuming that the annual cost of 1^st^-line therapy is US$128. (DOCX)

S8 Table Model outcomes: sensitivity analysis SL1 assuming that the annual cost of 2^nd^-line ART is US$210. (DOCX)

S9 Table Model outcomes: sensitivity analysis SL2 assuming that the annual cost of 2^nd^-line ART is US$140. (DOCX)

S10 Table Model outcomes: sensitivity analysis SL3 assuming that the annual cost of 2^nd^-line ART is US$350. (DOCX)

S11 Table Model outcomes: sensitivity analysis DI1 assuming no discounting. (DOCX)

S1 Text Technical description of the definitions related to the cost-effectiveness analyses. (DOCX)

S2 Text Sensitivity analyses. (DOCX)
