# Supplementary material for: The Cost-Effectiveness of Monitoring Strategies for Antiretroviral Therapy of HIV Infected Patients in Resource-Limited Settings: Software Tool
Source: PLoS One. 2015 Mar 20;10(3):e0119299. doi: 10.1371/journal.pone.0119299 (PMC4368574; doi:10.1371/journal.pone.0119299)
Supplement: S1 Table — (DOCX) [file pone.0119299.s002.docx]

**S1 Table. List of sensitivity analyses.**

| **Analysis** | **Varied input value** |
| --- | --- |
| VL1 | Cost of VL test: US$7 |
| VL2 | Cost of VL test: US$5 |
| VL3 | Cost of VL test: US$15 |
| CD1 | Cost of CD4 test: US$2 |
| FL1 | Cost of 1^st^-line ART: US$55/year |
| FL2 | Cost of 1^st^-line ART: US$128/year |
| SL1 | Cost of 2^nd^-line ART: US$210/year |
| SL2 | Cost of 2^nd^-line ART: US$140/year |
| SL3 | Cost of 2^nd^-line ART: US$350/year |
| DI1 | No discounting |

Only one variable was varied in each of the sensitivity analyses. Default parameters: cost of VL test US$10; cost of CD4 test US$5; cost of 1^st^-line ART US$99/year; cost of 2^nd^-line ART US$280/year.

VL, viral load; ART, antiretroviral therapy.
