# Supplementary material for: The Cost-Effectiveness of Monitoring Strategies for Antiretroviral Therapy of HIV Infected Patients in Resource-Limited Settings: Software Tool
Source: PLoS One. 2015 Mar 20;10(3):e0119299. doi: 10.1371/journal.pone.0119299 (PMC4368574; doi:10.1371/journal.pone.0119299)
Supplement: S4 Table — (DOCX) [file pone.0119299.s005.docx]

**S4 Table. Model outcomes: sensitivity analysis VL3 assuming that the cost of a viral load test is US$15.**

| **Strategy** | **No 2^nd^-l.** | **Clinical** | **CD4 monitoring** | | | | | **POC-VL monitoring** | | | **Lab-VL monitoring** | | |
| --- | --- | --- | --- | --- | --- | --- | --- | --- | --- | --- | --- | --- | --- |
|  | **1.1** | **2.1** | **3.1** | **3.2** | **3.3** | **3.4** | **3.5** | **4.1** | **4.2** | **4.3** | **5.1** | **5.2** | **5.3** |
| **Life-years** |  |  |  |  |  |  |  |  |  |  |  |  |  |
| Healthy life-years left | 19.5 | 19.5 | 19.5 | 19.5 | 19.5 | 19.5 | 19.5 | 19.5 | 19.5 | 19.5 | 19.5 | 19.5 | 19.5 |
| Life-years on 1^st^-line ART | 14.3 | 13.7 | 13.3 | 13.3 | 13.2 | 13.2 | 13.6 | 12.7 | 12.7 | 12.5 | 12.8 | 12.7 | 12.7 |
| Life-years on 2^nd^-line ART | 0.0 | 0.8 | 1.3 | 1.2 | 1.3 | 1.3 | 0.9 | 1.8 | 1.9 | 2.0 | 1.8 | 1.9 | 1.9 |
| Life-years without symptoms | 13.6 | 13.8 | 13.8 | 13.8 | 13.8 | 13.8 | 13.8 | 13.9 | 13.9 | 13.9 | 13.9 | 13.9 | 13.9 |
| Life-years with symptoms | 0.7 | 0.7 | 0.7 | 0.7 | 0.7 | 0.7 | 0.7 | 0.7 | 0.7 | 0.7 | 0.7 | 0.7 | 0.7 |
| Life-years lost to HIV | 5.2 | 5.1 | 5.0 | 5.0 | 5.0 | 5.0 | 5.0 | 5.0 | 4.9 | 5.0 | 5.0 | 5.0 | 4.9 |
| Disability-weighted life-years | 2.1 | 2.1 | 2.1 | 2.1 | 2.1 | 2.1 | 2.1 | 2.1 | 2.1 | 2.1 | 2.1 | 2.1 | 2.1 |
| ***DALYs lost to HIV*** | ***7.3*** | ***7.2*** | ***7.1*** | ***7.1*** | ***7.1*** | ***7.1*** | ***7.1*** | ***7.1*** | ***7.1*** | ***7.1*** | ***7.1*** | ***7.1*** | ***7.1*** |
| **Costs** |  |  |  |  |  |  |  |  |  |  |  |  |  |
| Cost of 1^st^-line ART | 1419 | 1353 | 1313 | 1313 | 1306 | 1304 | 1347 | 1261 | 1254 | 1238 | 1263 | 1256 | 1255 |
| Cost of 2^nd^-line ART | 0 | 211 | 351 | 341 | 363 | 365 | 259 | 507 | 534 | 563 | 499 | 521 | 530 |
| Cost of diagnostic tests | 0 | 0 | 71 | 36 | 72 | 143 | 162 | 109 | 221 | 438 | 143 | 288 | 574 |
| ***Total costs*** | ***1419*** | ***1564*** | ***1735*** | ***1690*** | ***1741*** | ***1812*** | ***1767*** | ***1876*** | ***2009*** | ***2240*** | ***1905*** | ***2065*** | ***2360*** |
| **Cost-effectiveness** |  |  |  |  |  |  |  |  |  |  |  |  |  |
| ***CER compared to 1.1*** | ***l/e*** | ***1651*** | ***1877*** | ***1967*** | ***2243*** | ***2959*** | ***1945*** | ***2305*** | ***2633*** | ***4593*** | ***2546*** | ***3200*** | ***4230*** |
| ***ICER*** | ***l/e*** | ***1651*** | ***2123*** | ***w/d*** | ***s/d*** | ***s/d*** | ***2980*** | ***w/d*** | ***5371*** | ***s/d*** | ***s/d*** | ***s/d*** | ***s/d*** |

Please see Table 2 of the main text for a detailed description of all monitoring strategies.

POC-VL, point-of-care viral load; lab-VL, laboratory-based viral load; ART, antiretroviral therapy; DALY, disability-adjusted life-year; CER, cost-effectiveness ratio; ICER, incremental cost-effectiveness ratio; l/e, least expensive and least effective strategy; w/d, weakly dominated; s/d, strongly dominated. All costs are given in US$ and cost-effectiveness ratios in US$ per DALY averted.
