# Supplementary material for: The Cost-Effectiveness of Monitoring Strategies for Antiretroviral Therapy of HIV Infected Patients in Resource-Limited Settings: Software Tool
Source: PLoS One. 2015 Mar 20;10(3):e0119299. doi: 10.1371/journal.pone.0119299 (PMC4368574; doi:10.1371/journal.pone.0119299)
Supplement: S9 Table — (DOCX) [file pone.0119299.s010.docx]

**S9 Table. Model outcomes: sensitivity analysis SL2 assuming that the annual cost of 2^nd^-line ART is US$140.**

| **Strategy** | **No 2^nd^-l.** | **Clinical** | **CD4 monitoring** | | | | | **POC-VL monitoring** | | | **Lab-VL monitoring** | | |
| --- | --- | --- | --- | --- | --- | --- | --- | --- | --- | --- | --- | --- | --- |
|  | **1.1** | **2.1** | **3.1** | **3.2** | **3.3** | **3.4** | **3.5** | **4.1** | **4.2** | **4.3** | **5.1** | **5.2** | **5.3** |
| **Life-years** |  |  |  |  |  |  |  |  |  |  |  |  |  |
| Healthy life-years left | 19.5 | 19.5 | 19.5 | 19.5 | 19.5 | 19.5 | 19.5 | 19.5 | 19.5 | 19.5 | 19.5 | 19.5 | 19.5 |
| Life-years on 1^st^-line ART | 14.3 | 13.7 | 13.3 | 13.3 | 13.2 | 13.2 | 13.6 | 12.7 | 12.7 | 12.5 | 12.8 | 12.7 | 12.7 |
| Life-years on 2^nd^-line ART | 0.0 | 0.8 | 1.3 | 1.2 | 1.3 | 1.3 | 0.9 | 1.8 | 1.9 | 2.0 | 1.8 | 1.9 | 1.9 |
| Life-years without symptoms | 13.6 | 13.8 | 13.8 | 13.8 | 13.8 | 13.8 | 13.8 | 13.9 | 13.9 | 13.9 | 13.9 | 13.9 | 13.9 |
| Life-years with symptoms | 0.7 | 0.7 | 0.7 | 0.7 | 0.7 | 0.7 | 0.7 | 0.7 | 0.7 | 0.7 | 0.7 | 0.7 | 0.7 |
| Life-years lost to HIV | 5.2 | 5.1 | 5.0 | 5.0 | 5.0 | 5.0 | 5.0 | 5.0 | 4.9 | 5.0 | 5.0 | 5.0 | 4.9 |
| Disability-weighted life-years | 2.1 | 2.1 | 2.1 | 2.1 | 2.1 | 2.1 | 2.1 | 2.1 | 2.1 | 2.1 | 2.1 | 2.1 | 2.1 |
| ***DALYs lost to HIV*** | ***7.3*** | ***7.2*** | ***7.1*** | ***7.1*** | ***7.1*** | ***7.1*** | ***7.1*** | ***7.1*** | ***7.1*** | ***7.1*** | ***7.1*** | ***7.1*** | ***7.1*** |
| **Costs** |  |  |  |  |  |  |  |  |  |  |  |  |  |
| Cost of 1^st^-line ART | 1419 | 1353 | 1313 | 1313 | 1306 | 1304 | 1347 | 1261 | 1254 | 1238 | 1263 | 1256 | 1255 |
| Cost of 2^nd^-line ART | 0 | 106 | 175 | 171 | 181 | 182 | 129 | 253 | 267 | 282 | 249 | 260 | 265 |
| Cost of diagnostic tests | 0 | 0 | 71 | 36 | 72 | 143 | 156 | 73 | 147 | 292 | 107 | 216 | 431 |
| ***Total costs*** | ***1419*** | ***1458*** | ***1559*** | ***1519*** | ***1559*** | ***1630*** | ***1632*** | ***1587*** | ***1802*** | ***1812*** | ***1620*** | ***1733*** | ***1951*** |
| **Cost-effectiveness** |  |  |  |  |  |  |  |  |  |  |  |  |  |
| ***CER compared to 1.1*** | ***l/e*** | ***445*** | ***833*** | ***727*** | ***977*** | ***1584*** | ***1187*** | ***844*** | ***1111*** | ***2198*** | ***1051*** | ***1553*** | ***2393*** |
| ***ICER*** | ***l/e*** | ***445*** | ***w/d*** | ***w/d*** | ***s/d*** | ***s/d*** | ***s/d*** | ***1158*** | ***3186*** | ***s/d*** | ***s/d*** | ***s/d*** | ***s/d*** |

Please see Table 2 of the main text for a detailed description of all monitoring strategies.

POC-VL, point-of-care viral load; lab-VL, laboratory-based viral load; ART, antiretroviral therapy; DALY, disability-adjusted life-year; CER, cost-effectiveness ratio; ICER, incremental cost-effectiveness ratio; l/e, least expensive and least effective strategy; w/d, weakly dominated; s/d, strongly dominated. All costs are given in US$ and cost-effectiveness ratios in US$ per DALY averted.
