# Supplementary material for: The Cost-Effectiveness of Monitoring Strategies for Antiretroviral Therapy of HIV Infected Patients in Resource-Limited Settings: Software Tool
Source: PLoS One. 2015 Mar 20;10(3):e0119299. doi: 10.1371/journal.pone.0119299 (PMC4368574; doi:10.1371/journal.pone.0119299)
Supplement: S11 Table — (DOCX) [file pone.0119299.s012.docx]

**S11 Table. Model outcomes: sensitivity analysis DI1 assuming no discounting.**

| **Strategy** | **No 2^nd^-l.** | **Clinical** | **CD4 monitoring** | | | | | **POC-VL monitoring** | | | **Lab-VL monitoring** | | |
| --- | --- | --- | --- | --- | --- | --- | --- | --- | --- | --- | --- | --- | --- |
|  | **1.1** | **2.1** | **3.1** | **3.2** | **3.3** | **3.4** | **3.5** | **4.1** | **4.2** | **4.3** | **5.1** | **5.2** | **5.3** |
| **Life-years** |  |  |  |  |  |  |  |  |  |  |  |  |  |
| Healthy life-years left | 31.7 | 31.7 | 31.7 | 31.7 | 31.7 | 31.7 | 31.7 | 31.7 | 31.7 | 31.7 | 31.7 | 31.7 | 31.7 |
| Life-years on 1^st^-line ART | 21.6 | 20.3 | 19.7 | 19.7 | 19.6 | 19.5 | 20.2 | 18.8 | 18.8 | 18.5 | 18.9 | 18.8 | 18.8 |
| Life-years on 2^nd^-line ART | 0.0 | 1.5 | 2.3 | 2.3 | 2.4 | 2.4 | 1.8 | 3.3 | 3.4 | 3.6 | 3.2 | 3.3 | 3.4 |
| Life-years without symptoms | 20.2 | 20.6 | 20.7 | 20.7 | 20.7 | 20.7 | 20.7 | 20.9 | 20.9 | 20.8 | 20.8 | 20.9 | 20.9 |
| Life-years with symptoms | 1.4 | 1.3 | 1.3 | 1.3 | 1.3 | 1.3 | 1.3 | 1.2 | 1.3 | 1.2 | 1.3 | 1.2 | 1.2 |
| Life-years lost to HIV | 10.1 | 9.9 | 9.7 | 9.7 | 9.7 | 9.8 | 9.7 | 9.6 | 9.5 | 9.6 | 9.6 | 9.6 | 9.6 |
| Disability-weighted life-years | 3.3 | 3.2 | 3.3 | 3.3 | 3.3 | 3.3 | 3.3 | 3.3 | 3.3 | 3.3 | 3.3 | 3.3 | 3.3 |
| ***DALYs lost to HIV*** | ***13.3*** | ***13.1*** | ***12.9*** | ***13.0*** | ***13.0*** | ***13.0*** | ***12.9*** | ***12.9*** | ***12.8*** | ***12.9*** | ***12.9*** | ***12.9*** | ***12.8*** |
| **Costs** |  |  |  |  |  |  |  |  |  |  |  |  |  |
| Cost of 1^st^-line ART | 2142 | 2014 | 1951 | 1948 | 1938 | 1935 | 2002 | 1864 | 1856 | 1829 | 1869 | 1861 | 1860 |
| Cost of 2^nd^-line ART | 0 | 412 | 655 | 644 | 676 | 676 | 510 | 918 | 956 | 1005 | 901 | 929 | 941 |
| Cost of diagnostic tests | 0 | 0 | 108 | 55 | 110 | 217 | 235 | 119 | 225 | 445 | 165 | 330 | 219 |
| ***Total costs*** | ***2142*** | ***2426*** | ***2714*** | ***2647*** | ***2723*** | ***2828*** | ***2747*** | ***2893*** | ***3038*** | ***3278*** | ***2935*** | ***3120*** | ***3457*** |
| **Cost-effectiveness** |  |  |  |  |  |  |  |  |  |  |  |  |  |
| ***CER compared to 1.1*** | ***l/e*** | ***1485*** | ***1509*** | ***1544*** | ***1738*** | ***2226*** | ***1602*** | ***1687*** | ***1810*** | ***2766*** | ***1835*** | ***2154*** | ***2705*** |
| ***ICER*** | ***l/e*** | ***1485*** | ***1535*** | ***w/d*** | ***s/d*** | ***s/d*** | ***s/d*** | ***2700*** | ***2912*** | ***s/d*** | ***s/d*** | ***s/d*** | ***s/d*** |

Please see Table 2 of the main text for a detailed description of all monitoring strategies.

POC-VL, point-of-care viral load; lab-VL, laboratory-based viral load; ART, antiretroviral therapy; DALY, disability-adjusted life-year; CER, cost-effectiveness ratio; ICER, incremental cost-effectiveness ratio; l/e, least expensive and least effective strategy; w/d, weakly dominated; s/d, strongly dominated. All costs are given in US$ and cost-effectiveness ratios in US$ per DALY averted.
