# Supplementary material for: The Cost-Effectiveness of Monitoring Strategies for Antiretroviral Therapy of HIV Infected Patients in Resource-Limited Settings: Software Tool
Source: PLoS One. 2015 Mar 20;10(3):e0119299. doi: 10.1371/journal.pone.0119299 (PMC4368574; doi:10.1371/journal.pone.0119299)
Supplement: S1 Text — (DOCX) [file pone.0119299.s013.docx]

**S1 Text. Technical description of the definitions related to the cost-effectiveness analyses.**

***Disability-adjusted life years (DALYs).*** DALYs are defined as the sum of life-years lost to HIV (*YLL*) and life-years lived with disability (*YLD*), where:

- $YLL=\int_{0}^{A_{nat}-A_{0}} \left( 1+p \right)^{-t}dt-\int_{0}^{A_{true}-A_{0}} \left( 1+p \right)^{-t}dt$, where *A*_0_ is the age at ART start, *A_nat_* the age at simulated time of expected death without HIV, *A_true_* the age at simulated true death and *p* the rate of annual discounting
- $YLD=\int_{0}^{A_{true}-A_{0}} D\left( t \right)\left( 1+p \right)^{-t}dt$, where *A*_0_, *A_true_* and *p* are as before and *D*(*t*) the disability coefficient at time *t*

***Costs.*** Total costs (*C*) were consisted of ARVs, visits and diagnostic tests:

$$C=\int_{0}^{A_{switch}-A_{0}} C_{first}\left( 1+p \right)^{-t}dt+\int_{A_{switch}-A_{0}}^{A_{true}-A_{0}} C_{second}\left( 1+p \right)^{-t}dt+\sum_{i\in V} C_{visit}\left( 1+p \right)^{-\left( t\left( i \right)-A_{0} \right)}+\sum_{i\in M_{VL}} C_{VL}\left( 1+p \right)^{-\left( t\left( i \right)-A_{0} \right)}+\sum_{i\in M_{CD4}} C_{CD4}\left( 1+p \right)^{-\left( t\left( i \right)-A_{0} \right)}$$

where *A*_0_, *A_true_* and *p* are as before, *A_switch_* is the age at switching to 2^nd^-line therapy, *C_first_* is the annual cost of 1^st^-line ART, *C_second_* the annual cost of 2^nd^-line ART, *C_visit_* the cost of a clinic appointment, *C_VL_* the cost of a viral load measurement, *C_CD4_* the cost of a CD4 measurement, *V*, *M_VL_* and *M_CD4_* the sets of clinic appointments, viral load measurements and CD4 measurements, respectively, and *t*(*i*) the age of the patient at visit or measurement *i*. The costs of visits were excluded from the presentation of the results in the manuscript, but can be included in the Excel table.

***Cost-effectiveness.*** We used the incremental cost-effectiveness ration (ICER) as the main measure for cost-effectiveness. ICER of a more effective and more expensive strategy compared to a less expensive and less effective strategy was defined as

$ICER=\frac{C_{1}-C_{0}}{{DALY}_{0}-{DALY}_{1}}$ ,

where *C*_1_ and *DALY*_1_ are the costs and lost DALYs in the more expensive strategy and *C*_0_ and *DALY*_0_ the costs and lost DALYs in the cheaper strategy (i.e. the ICER gives the cost of averting one DALY). We present the cost-effectiveness ratios of each strategy 2.1 to 5.3 compared to strategy 1.1, as well as ICERs between all consecutive strategies in the sequence of non-dominated strategies, starting from the cheapest and least effective strategy going towards more expensive and more effective strategies. Domination was defined using the standard terms of cost-effectiveness analysis. A *strongly dominated* strategy means that this strategy is more expensive than at least one more effective strategy. A *weakly dominated* strategy means that the strategy is not strongly dominated, but that a linear combination of one cheaper and one more expensive strategy would result in similar effectiveness with lower costs.
