# Supplementary material for: The Cost-Effectiveness of Monitoring Strategies for Antiretroviral Therapy of HIV Infected Patients in Resource-Limited Settings: Software Tool
Source: PLoS One. 2015 Mar 20;10(3):e0119299. doi: 10.1371/journal.pone.0119299 (PMC4368574; doi:10.1371/journal.pone.0119299)
Supplement: S2 Text — (DOCX) [file pone.0119299.s014.docx]

**S2 Text. Sensitivity analyses**

We present a total of 10 sensitivity analyses (S1 Table). We varied the costs of viral load tests from US$10 to US$7 (VL1), US$5 (VL2) and US$15 (VL3), assuming however that POC and laboratory-based tests would cost the same. Reduction of CD4 test cost to US$2 from US$5 was also explored (CD1). Costs of 1^st^-line (FL1: US$55; or FL2: US$128 instead of US$99 per year) and 2^nd^-line antiretrovirals (SL1: US$210; SL2: US$140; or SL3: US$350 instead of US$280 per year) were also varied. We also present a sensitivity analysis with no discounting (DI1). The full results of all sensitivity analyses are presented only in the scenario where the failure rates are identical in all strategies. We do not present combinations of varying two or more parameters simultaneously: such analyses can however be performed by the user with the Excel tool easily. Cost-effectiveness ratios of Strategy 4.2 (POC VL monitoring every 12 months) compared with Strategy 2.1 (clinical monitoring) are also presented in Table 6 of the main text, for both scenarios, A (identical failure rates in both strategies) and B (failure rate is twice as high in Strategy 4.2 compared with Scenario 2.1).

In the first set of sensitivity analyses we varied the unit cost of the viral load test. Reducing the cost to US$7 (VL1; S2 Table) or US$5 (VL2; S3 Table) improved the cost-effectiveness of viral load monitoring and Strategy 3.1 became dominated. If the cost of viral load test was US$5 (VL2), i.e. the same as of a CD4 test, the viral load monitoring strategies were still more expensive due to the increased use of 2^nd^-line ART. Increasing the cost of the viral load test to US$15 (VL3) correspondingly lead to viral load monitoring being less cost-effective (S4 Table): the pattern of domination was the same as in the main analysis, but the cost-effectiveness ratios were higher, e.g. with Strategy 4.2 the prevention of one DALY cost US$4902 compared with Strategy 3.1.

When the cost of viral load test remained at US$10 and the CD4 test was reduced to US$2 (CD1), targeted viral load monitoring became increasingly cost-effective (S5 Table): targeted viral load monitoring (Strategy 3.5) was the least expensive non-dominated strategy with 2^nd^-line ART, and the ICER compared with no 2^nd^-line (Strategy 1.1) was US$1430/DALY averted. Twelve-monthly routine POC viral load monitoring (4.2) remained also non-dominated, but the ICER compared with targeted viral load monitoring (3.5) was above US$5000/DALY averted.

In the sensitivity analyses where the annual costs of 1^st^- or 2^nd^-line ARVs were varied, the ratio of 1^st^- and 2^nd^-line ART costs played the highest role. Reducing the 1^st^-line cost to U$55 (FL1; S6 Table) or increasing the 2^nd^-line cost to US$350 (SL3; S10 Table) means that 2^nd^-line becomes clearly more expensive than 1^st^-line. This increased the cost-effectiveness ratios of all 2^nd^-line strategies compared with no 2^nd^-line (1.1) to at least about US$2000/DALY averted. In these scenarios, clinical monitoring (2.1), targeted viral load monitoring (3.5) and 12-monthly routine POC-viral load monitoring (4.2) were not dominated. However, if 1^st^-line ART cost was increased to US$128/year (FL2; S7 Table) or 2^nd^-line ART cost reduced to US$210/year (SL1; S8 Table) or US$140/year (SL2; S9 Table), the costs of the regimens became closer to each other. The cost-effectiveness of all 2^nd^-line strategies was improved, in particular the routine viral load monitoring strategies. Targeted viral load monitoring (3.5) became dominated and 24-monthly routine POC viral load monitoring (4.1) non-dominated.

In the last sensitivity analysis we removed discounting (DI1; S11 Table). Compared with the main analysis, targeted viral load monitoring (3.5) became dominated and 24-monthly viral load monitoring (4.1) non-dominated.
